# Lapatinib Induces Autophagy, Apoptosis and Megakaryocytic Differentiation in Chronic Myelogenous Leukemia K562 Cells

**DOI:** 10.1371/journal.pone.0029014

**Published:** 2011-12-22

**Authors:** Huey-Lan Huang, Yu-Chieh Chen, Yu-Chuen Huang, Kai-Chien Yang, Hsin yi Pan, Shou-Ping Shih, Yu-Jen Chen

**Affiliations:** 1 Department of Bioscience Technology, College of Health Science, Chang Jung Christian University, Tainan, Taiwan; 2 Department of Medical Research, China Medical University Hospital, Taichung, Taiwan; 3 Graduate Institute of Chinese Medical Science, College of Chinese Medicine, China Medical University, Taichung, Taiwan; 4 Department of Medical Research, Mackay Memorial Hospital, Taipei, Taiwan; 5 Department of Radiation Oncology, Mackay Memorial Hospital, Taipei, Taiwan; 6 Institute of Traditional Medicine, School of Medicine, National Yang-Ming University, Taipei, Taiwan; 7 Institute of Pharmacology, Taipei Medical University, Taipei, Taiwan; University of Chicago, United States of America

## Abstract

Lapatinib is an oral, small-molecule, dual tyrosine kinase inhibitor of epidermal growth factor receptors (EGFR, or ErbB/Her) in solid tumors. Little is known about the effect of lapatinib on leukemia. Using human chronic myelogenous leukemia (CML) K562 cells as an experimental model, we found that lapatinib simultaneously induced morphological changes resembling apoptosis, autophagy, and megakaryocytic differentiation. Lapatinib-induced apoptosis was accompanied by a decrease in mitochondrial transmembrane potential and was attenuated by the pancaspase inhibitor z-VAD-fmk, indicating a mitochondria-mediated and caspase-dependent pathway. Lapatinib-induced autophagic cell death was verified by LC3-II conversion, and upregulation of Beclin-1. Further, autophagy inhibitor 3-methyladenine as well as autophagy-related proteins Beclin-1 (ATG6), ATG7, and ATG5 shRNA knockdown rescued the cells from lapatinib-induced growth inhibition. A moderate number of lapatinib-treated K562 cells exhibited features of megakaryocytic differentiation. In summary, lapatinib inhibited viability and induced multiple cellular events including apoptosis, autophagic cell death, and megakaryocytic differentiation in human CML K562 cells. This distinct activity of lapatinib against CML cells suggests potential for lapatinib as a therapeutic agent for treatment of CML. Further validation of lapatinib activity in vivo is warranted.

## Introduction

Epidermal growth factor receptors (EGFR or ErbB/Her) belong to the receptor tyrosine kinase superfamily. The EGFR subclass is made up of four closely related members: EGFR/ErbB1/Her1, ErbB2/Her2/Neu, ErbB3/Her3, ErbB4/Her4 [1], [2], [3]. Formation of homodimers or heterodimers of ErbB receptors is triggered after binding to EGF-related growth factors. Upon activation, autophosphorylation of tyrosine residues within the cytoplasmic domains of EGFR/ErbB receptors trigger intracellular signaling pathways, such as the phosphatidylinosithol-3-kinase (PI3K) pathway, the Shc- and/or Grb2-mediated mitogen-activated protein kinase (MAPK)-ERK1/2 pathway(s), the protein kinase C (PKC) pathway, and other pathways involved in proliferation response.

Due to the pivotal roles of aberrant EGFR signaling pathways in the development of different kinds of malignant human cancers, the receptor tyrosine kinase superfamily is well-studied. Overexpression of ErbB2 is found in about 30% of breast cancer patients and is correlated with poor prognosis [1], [2], [3], [4]. Among the ErbB receptors, ErbB2 lacks its own ligands; therefore, ErbB2 forms heterodimers with EGFR, ErbB3 or ErbB4, or even with other family members, such as Insulin-like growth factor-1 receptor (IGF-1R) [5], [6]. Findings such as these suggest that the ErbBs may be good molecular targets for various malignancies, including breast cancer.

Lapatinib (Tykerb or GW-572016, GlaxoSmithKline) is a small-molecule, tyrosine kinase inhibitor which targets both ErbB1 and ErbB2 [2], [3]. Due to its specificity to EGFR family members, applicability to oral administration, and seemingly few adverse effects, lapatinib has received considerable attention and is undergoing clinical trials for treatment of various solid tumors, including breast, head and neck, vulva, colon, prostate, and stomach [7], [8], [9], [10], [11], [12], [13]. Lapatinib shows promise as a therapy in combination with capecitabine for patients with Her2-overexpressing advanced or metastatic breast cancer that fails to respond to anthracycline, taxane, and the anti-Her2 monoclonal antibody transtuzumab [2], [3]. As yet, however, no hematological malignancies have been included in laboratory or clinical investigations of lapatinib.

To date, agents possessing cytotoxicity, targeted therapeutic activity and differentiation-inducing capacity have proven most effective for treating leukemia, the most common hematological cancer [14]. Chronic myelogenous leukemia (CML) is a clonal disease characterized by the presence of the Philadelphia chromosome and resultant BCR-Abl gene fusion with constitutively active tyrosine kinase in >90% of patients. CML cells producing BCR-Abl fusion protein, such as the K562 cell line, are important experimental models because they allow the assessment of multiple cellular and molecular events simultaneously, including various modes of cell death [15], [16], differentiation toward the erythroid/macrophage/megakaryocyte lineages [14], [16], [17], [18], [19], [20], and downegulation of BCR-Abl tyrosine kinase [21].

In recent years, molecular target-based cancer therapy has been successfully applied to improve the efficacy and ameliorate the adverse effects of several conventional chemotherapeutic drugs. The pharmacological activities of novel targeted therapeutics may not be limited to currently recognized targets. For example, the EGFR inhibitor erlotinib overcame drug resistance [22], and PKC412 induced megakaryocytic differentiation in K562 cells [19]. In this study, we evaluated the effect of lapatinib on cell death and differentiation, and investigated its mechanism of action in human CML K562 cells. We found that lapatinib induced multiple cellular events simultaneously including apoptosis, autophagic cell death, and megakaryocytic differentiation in human CML K562 cells. Apoptosis was likely induced by a caspase-dependent pathway and autophagic cell death was likely induced via an ATG6-dependent pathway.

## Methods

### Cell culture and drug treatments

CML-derived K562 and MEG-01, acute myeloblastic leukemia (AML)-derived HL-60, and acute promyelocytic leukemic NB4 cells were cultured in Roswell Park Memorial Institute (RPMI) 1640 medium supplemented with 10% fetal bovine serum (Gibco, Grand Island, NY), 100 IU/ml of penicillin, 100 mg/ml of streptomycin (Gibco), and 50-µM β-mercaptoethanol. Fresh blood from healthy donors was used for isolation of primary CD14^+^ mononuclear cells (MNC) by Ficoll-Paque PLUS density gradient (Amersham Biosciences, Sunnyvale, CA) and positive selection using CD14 MicroBeads (MACS Miltenyi Biotech, Auburn, CA) according to manufacturer's instructions. The use of human peripheral blood leukocytes to isolate monocytes was approved by the institutional review board of Mackay Memorial Hospital, Taipei, Taiwan. Both human CD14^+^ monocytes and mouse bone marrow cells isolated from femur were cultured in RPMI 1640 medium supplemented with 10% serum. Lapatinib was dissolved in dimethyl sulfoxide (DMSO, Sigma Chemical Company, St. Louis, MO) as a 1,000-fold stock solution. K562 cells were either left untreated, or incubated with DMSO as vehicle control and various concentrations of lapatinib for 1–3 days as indicated. For 1.25- or 2.5-mM 3-methyladenine (3-MA, Sigma) co-treatment experiments, a 20-mM stock solution of 3-MA was made up in culture medium. To test the role of caspases, K562 cells were treated with lapatinib alone or co-treated with both lapatinib and 20 µM of the pancaspase inhibitor z-VAD-fmk (R&D, Minneapolis, MN), and dissolved in DMSO as a 1,000-fold stock solution. For some experiments, 1-µM 12-*O*-Tetradecanoylphorbol 13-acetate (TPA, Sigma) treatment was used as the positive control for megakaryocytic differentiation of the K562 cells. For observation of morphology, cells were treated with drugs for 3 days and then attached on slides using cytospin apparatus (CytoSpin, Thermo Fisher Scientific), stained with Liu's stain according to the manufacturer's instructions and observed microscopically.

### Growth inhibition analysis

To assess cell growth kinetics, viable cells were counted using the trypan blue (Gibco) dye exclusion method. K562 cells were treated and counted on days 1, 2, and 3. The viability of the cells was also measured using the tetrazolium (MTT) assay (Sigma) or 3-(4,5-dimethylthiazol-2-yl)-5-(3-carboxymethoxyphenyl)-2-(4-sulfophenyl)-2H-tetrazolium, inner salt (MTS) assay (Promega, Madison, WI). For the MTT assay, 1-mg/mL MTT was added to the culture medium and the cells were incubated at 37°C for 4 h, then an equal volume of acid isopropanol (0.04 M HCl in isopropanol) was added to dissolve the MTT dye inside the viable cells. The absorbance was measured at 570 nm using an enzyme-linked immunosorbent assay reader. For some experiments MTS viability assay was conducted according to the manufacturer's instructions. The optical density (OD) value of control cells (DMSO vehicle treated) was designated as 100% viability. IC_50_ values were calculated by GraphPad Prism 4 software (GraphPad Software, San Diego, CA) from MTT assay data at day 3.

### Detection of apoptotic or dead cells by flow cytometry

K562 cells arrested at the sub G1 phase (hypodiploid or with DNA laddering) were detected using a cell-cycle detection assay in which the cells were resuspended in propidium iodide (PI, Sigma)-containing hypotonic buffer (0.1% sodium citrate, 0.1% Triton X-100, and 5 µg/ml PI) as previously described [15], [16]. The DNA content of the cells was measured by flow cytometry (FACSCalibur, Becton Dickenson, Mountain View, CA), and the percentage of sub G1 cells was calculated using FlowJo software (Tree Star, Ashland, OR). For the annexin V apoptosis assay, K562 cells were collected and resuspended in binding buffer containing annexin V-fluorescein isothiocyanate (FITC) (Clontech, Mountain View, CA) and PI; The percentage of apoptotic cells was measured by flow cytometry with FSC vs. SSC plot (gated on live cell population) using FlowJo software (excluded cell debris with PI staining). For detection of the mitochondrial transmembrane potential of cells, 3,3′-dihexyloxacarbocyanine iodide (DiOC6_(3)_) (Molecular Probes, Eugene, OR) was used and detected by flow cytometry according to manufacturer's instructions. For detection of CD61^+^ cells, cells were stained with FITC-conjugated CD61 antibody (BD Biosciences Pharmingen, San Diego, CA) and 7-AAD viability staining to exclude dead cells, and then measured by flow cytometry.

### Western blot analysis

After treatment, cells were collected and dissolved in lysis buffer (50-mM Tris-HCl [pH 7.4], 150-mM NaCl, 5-mM EDTA, and 10% glycerol) with protease inhibitors (Roche Biochemicals, Indianapolis, IN). Cell lysates were then subjected to 10% sodium dodecylsulfate-polyacrylamide gel electrophoresis, and immunoblotted with the following antibodies: actin (Sigma), LC3 (Abgent, San Diego, CA), ATG7 (Santa Cruz Biochemicals, Santa Cruz, CA), Beclin-1 (or ATG6) (Cell Signaling, Beverly, MA), and ATG12 (Cell Signaling) for detection of ATG5-ATG12 conjugates.

### RNA-mediated gene knockdown

A lentivirus-based short hairpin RNA (shRNA) expression system (The RNAi Consortium [TRC]) was used to knock down gene expression of ATG5, ATG7 and Beclin-1. Galactosidase beta 1 (LacZ), and red fluorescent protein (RFP) specific constructs were used as non-targeting shRNA controls. We obtained all pLKO.1-shRNA constructs from the National RNAi Core Facility, Academia Sinica, Taipei, Taiwan. The following plasmids were used: TRCN0000151963 (ATG5), TRCN0000007587 (ATG7), TRCN0000033549 (Beclin-1, #1), TRCN0000033552 (Beclin-1, #2), TRCN0000072237 (LacZ), and TRCN0000072216 (RFP). Lentivirus productions and lentiviral spin infections were performed according to protocols provided by TRC. After puromycin selection, cells were treated with lapatinib and viability was assayed, as described above.

## Results

### Effects of lapatinib on viability and morphology of human CML K562 cells

**Figure 1:**
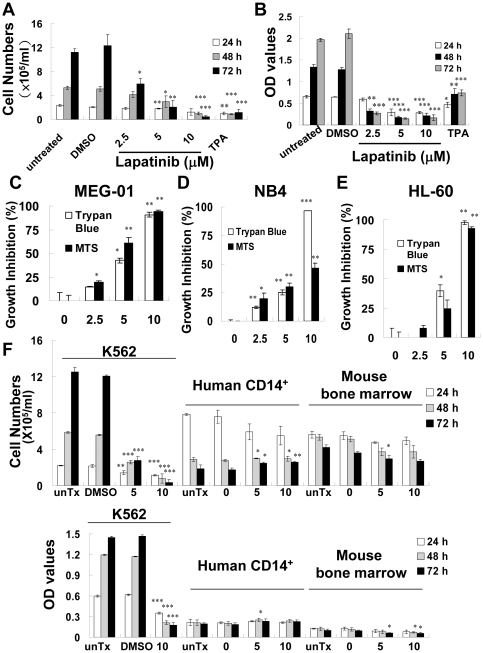
Inhibition of leukemia cell proliferation by lapatinib. K562 CML cells were left untreated or were treated with 0.1% dimethyl sulfoxide (DMSO, vehicle), DMSO with different doses of lapatinib (2.5, 5, or 10 µM), or 12-*O*-Tetradecanoylphorbol 13-acetate (TPA) 10 µM for 1 to 3 days as indicated. (A) Cell numbers for each treatment were counted using trypan-blue dye exclusion assay. (B) Relative numbers of viable cells were detected using MTT assay. (C–F) Inhibition of cell proliferation by lapatinib in leukemia cell lines, but not in primary CD14^+^ mononuclear or bone marrow cells. 1×10^5^/ml CML MEG-01 (C), AML NB4 (D), HL-60 cells (E), K562, or 5×10^5^/ml human CD14^+^ mononuclear and mouse bone marrow cells (F) were untreated (unTx), treated with DMSO vehicle (0), or DMSO with different doses of lapatinib (µM) for 2 (C), 3 (D–E), or 1–3 days (F). The raw data (B and F) or relative percentage of growth inhibition (C–E) were assessed and calculated using both trypan-blue dye exclusion and MTS assays (C–E) or MTT assays (F) as indicated in each figure as described in (A–B). Optical density (OD) values from DMSO-treated control cells were used as a standard (0% cell death), and the relative percentage of growth inhibition was calculated using the following method: [(mean OD values from DMSO-treated cells – mean OD values from drug-treated cells)/mean OD values from DMSO-treated cells]×100. The data are expressed as the mean ± SEM. *P<0.05, **P<0.01, ***P<0.001 (*t*-test) between lapatinib-treated and DMSO control cells.

Cell viability was evaluated using trypan blue dye exclusion assay. Lapatinib reduced the number of viable K562 cells in a dose-and time-dependent manner (Fig. 1A). This inhibitory activity was verified using an MTT assay which showed a half-maximal inhibitory concentration (IC_50_) of 1.49 µM for lapatinib in K562 cells (Fig. 1B). To compare this result with the effect of lapatinib on other leukemia cell lines, CML-derived MEG-01, and AML-derived HL-60 and NB4 cells were tested. A similar pattern of cytotoxicity was noted in all the cell lines tested (Figs. 1C–1E). In contrast, lapatinib was less toxic to normal, primary human CD14^+^ monocytes and mouse bone marrow cells (Fig. 1F).

### Effect of lapatinib on apoptosis

**Figure 2:**
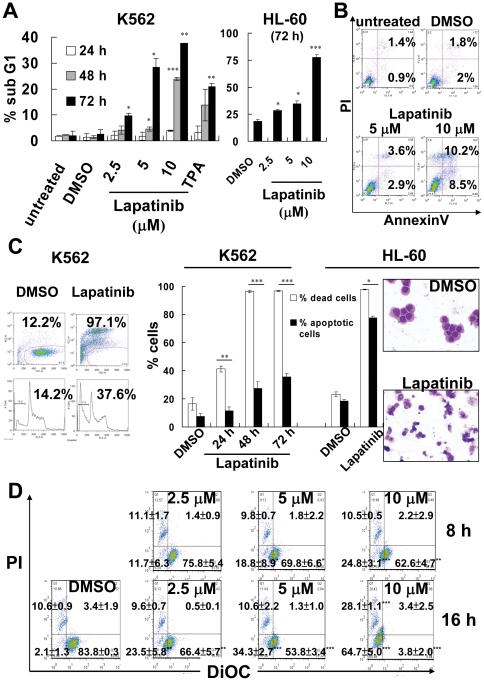
Induction of apoptosis by lapatinib in K562 and HL-60 cells. (A) K562 or HL-60 cells were left untreated or treated with various concentrations of lapatinib or TPA as indicated for 1–3 days. Cells were collected and resuspended in propidium iodide (PI)-containing hypotonic buffer, and then the percentage of apoptotic cells with DNA ladders (hypodiploid cells) was analyzed by flow cytometry. The data are expressed as means ± SEMs. (B) K562 cells were left untreated or treated with lapatinib for 3 days; cells were then collected, resuspended in both AnnexinV and PI containing buffer, and analyzed by flow cytometry. The percentages of PI(+)/annexin(+) or PI(−)/annexin(+) cells are indicated in each figure. (C) Induction of both apoptotic and non-apoptotic cell death by lapatinib in K562 cells. After DMSO or 10 µM lapatinib treatment for 1–3 days for K562, or 3 days for HL-60, cells were split into two tubes and resuspended in PI-containing phosphate buffered saline (upper panel in left figure, total dead cells) or PI-containing hypotonic buffer (lower panel in left figure, apoptotic cells), respectively, for simultaneously detecting total dead cells without intact plasma membranes or apoptotic cells as described in (A). The graph in the right panel represents the data as: the percentage of total dead cells (empty bars) and the percentages of apoptotic cells (solid bars). After drug treatment for 3 days, HL-60 cells were attached on slides using cytospin apparatus and observed after staining with Liu's stain (right panel of 2C). (D) After DMSO, 2.5, 5 or 10 µM lapatinib treatment for 8 or 16 h, K562 cells were stained with both PI and DiOC6_(3)_. The mitochondrial transmembrane potential of the cells was analyzed by flow cytometry. *P<0.05, **P<0.01, ***P<0.001 (*t*-test) between treated and DMSO control cells.

**Figure 3:**
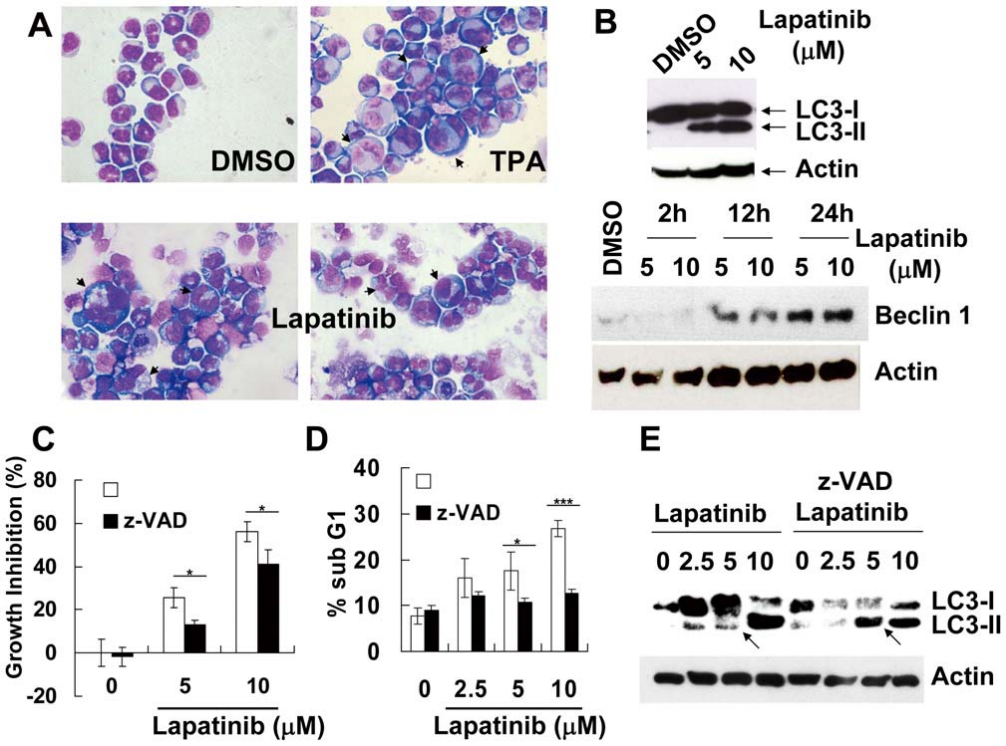
Induction of autophagy in lapatinib-treated K562 cells. (A) Induction of morphological changes by lapatinib in K562 cells. After drug treatment for 3 days, cells were attached on slides using cytospin apparatus and observed microscopically after staining with Liu's stain. Arrows indicate cells with giant contours, characteristic of megakaryocytic differentiation. (B) Expression profiles of autophagic factors in lapatinib-treated K562 cells. K562 cell lysates were prepared 3 days (for LC3) or as indicated (for Beclin-1) after DMSO, 5, or 10 µM lapatinib exposure, then, the amounts of LC3, Beclin-1, and actin were examined by immunoblotting using antibodies against the respective proteins. LC3-II is an autophagic marker. (C–E) Abrogation of lapatinib-induced sub G1, but not growth inhibition, by pancaspase inhibitor z-VAD-fmk. After K562 cells were treated with lapatinib alone or lapatinib plus 20 µM z-VAD-fmk for 48 h, growth inhibition (C), percentage of sub G1 cells (D), or LC3I to LC3II conversion (LC3I/II) (E) was assessed by MTS assay, flow cytometry, or immunoblotting as described in Fig. 2 or Fig. 3B. Increased LC3II formation is indicated by arrows. *P<0.05, ***P<0.001 (*t*-test).

Cell-cycle analysis of DNA hypodipolid sub G1 cell components (Fig. 2A–2C) and flow cytometric analysis of externalized phosphatidylserine (Fig. 2B) suggested that lapatinib induced apoptosis in both K562 and HL-60 cells. After exposure for 16 h, lapatinib reduced the mitochondrial transmembrane potential prior to reduction of cell viability (Fig. 2D), indicating involvement of the mitochondria-mediated apoptotic pathway. Lapatinib reduced viability and induced distinct morphological alterations in human CML K562 cells. Intriguingly, morphological observation revealed multiple morphological cellular events at the effective concentration, including chromatin condensation, formation of apoptotic bodies, extensive intra-cytoplasm vesicles, and multi-nucleated giant cells (Fig. 3A). These changes resembled changes in K562 cells treated with TPA, a drug known to induce K562 cells to differentiate towards the megakaryocytic lineage [16], [20]. Co-treatment with the pancaspase inhibitor z-VAD-fmk partially blocked lapatinib-induced inhibition of viability and apoptosis induction, suggesting that lapatinib activates both caspase-dependent and caspase-independent cell death pathways (Figs. 3C and 3D). Interestingly, at conditions that reduced viability more than 95%, fewer than 40% of the K562 cells were positive for apoptosis (Fig. 2C), in contrast, 80% of HL-60 cells were positive for apoptosis after lapatinib treatment (right panel of Fig. 2C). The morphological features of lapatinib-treated HL-60 cells correlated with the high percentage of apoptotic cells. High levels of dead cells were detected at days 1–3, indicating that the reduction in K562 cell numbers after lapatinib treatment is not due to growth arrest or induction of apoptosis at later time points. This raises the possibility that other, non-apoptotic modes of cell death might be induced by lapatinib in K562 cells.

### Lapatinib induces autophagic cell death in K562 cells via an ATG6-dependent pathway

**Figure 4:**
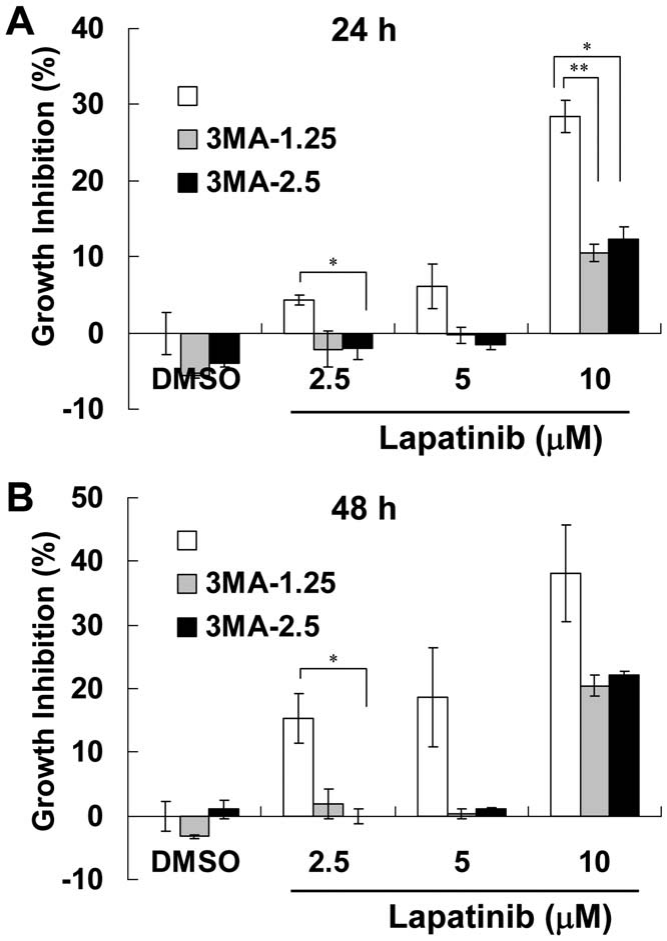
Rescue of K562 cells from lapatinib-induced cytotoxicity by the autophagy inhibitor 3-methyladenine (3-MA). K562 cells were left untreated or were treated with various concentrations of lapatinib in the presence or absence of 1.25-mM or 2.5-mM 3-MA for 24 (A) or 48 h (B) as indicated in each figure. Relative amounts of viable cells were detected using the MTS assay, and the relative percentage of growth inhibition was calculated as described in Fig. 1. *P<0.05, **P<0.01 (*t*-test).

Because lapatinib induced numerous small vesicles in the cytoplasm of a large proportion of treated cells (Fig. 3A), we investigated the conversion of microtubule-associated protein 1 light chain 3-I (LC3-I) to the phoshatidylethanolamine-conjugated form, LC3 (LC3-II), an indicator of autophagy [16], [20]. Treatment of the cells with lapatinib induced LC3-II formation in a dose-dependent manner (Fig. 3B), similar to LC3-II induction patterns in serum-starved K562 cells (data not shown). To investigate whether lapatinib-induced autophagy caused cell death, the effect of 3-methyladenine (3-MA), which inhibits the sequestration of the autophagy process, was tested. Pretreatment of cells with 3-MA attenuated the lapatinib-mediated reduction of viability (Fig. 4). This suggested that lapatinib-induced autophagy is a mode of cell death.

**Figure 5:**
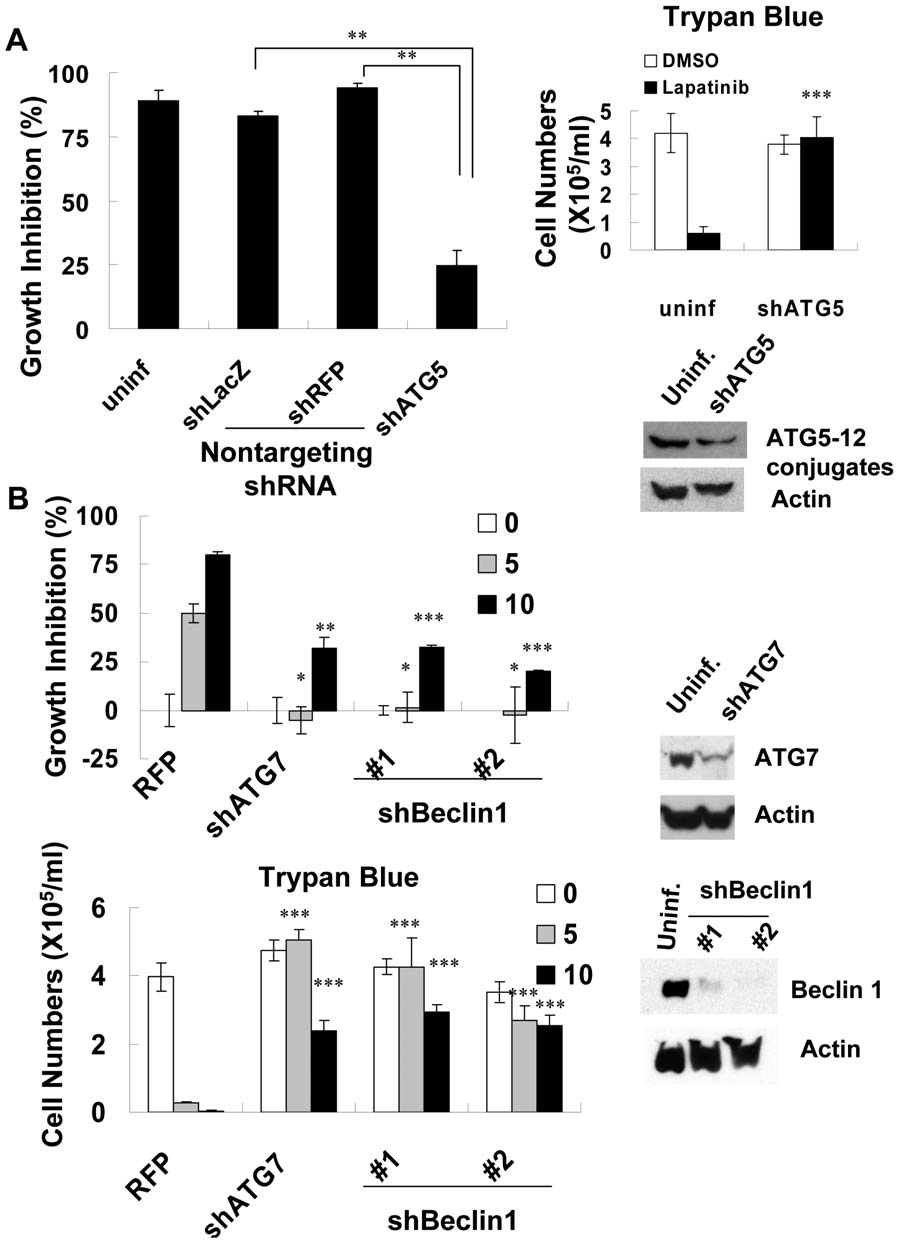
Protection of K562 cells from lapatinib-induced cytotoxicity by knockdown of autophagy-related proteins. After transduction with shRNA expression lentivirus as indicated in each figure, K562 cells were selected and kept in puromycin-containing medium. Cells were treated with DMSO or lapatinib for 72 (A) or 48 h (B), and then the relative percentages of growth inhibition were detected using the MTS assay (A, left panel and B, upper panel) or trypan blue exclusion assay (A, right panel and B, lower panel) and calculated as described in Fig. 1. Knockdown efficiency of ATG7, beclin-1, and ATG5-12 conjugates or the loading control, actin, were examined by immunoblotting using antibody against the respective proteins as described in Fig. 3. *P<0.05, **P<0.01, ***P<0.001 (*t*-test).

We further examined the expression level of Beclin-1 (ATG6 protein), the mammalian homolog of yeast autophagy protein ATG8, by immunoblot. Lapatinib induced expression of Beclin-1 in K562 cells in a dose dependent manner (Fig. 3B). To elucidate the role of Beclin-1 in the lapatinib-treated cells, we knocked down expression of Beclin-1 and autophagy-related proteins ATG7 and ATG5 after transduction with a shRNA expression lentivirus system (Fig. 5). Specific knockdown of Beclin-1, ATG7, and ATG5 mRNA, but not the non-targeting shRNA, rescued the cells from lapatinib-mediated cell death, indicating that lapatinib induced autophagy via an ATG6-dependent pathway (Fig. 5).

To clarify the role of caspases in lapatinib-induced autophagy, we co-treated K562 cells with pancaspase inhibitor z-VAD-fmk and lapatinib. Under conditions optimized for attenuation of lapatinib-induced apoptosis, z-VAD-fmk augmented the conversion of LC3-I to LC3-II (Fig. 3E). These data suggest that the autophagic cell death-induced by lapatinib could be caspase independent in K562 cells. Thus, it is probable that lapatinib induced autophagic cell death in K562 cells occurs via an ATG6-dependent and caspase-independent pathway.

### Effect of lapatinib on megakaryocytic differentiation

**Figure 6:**
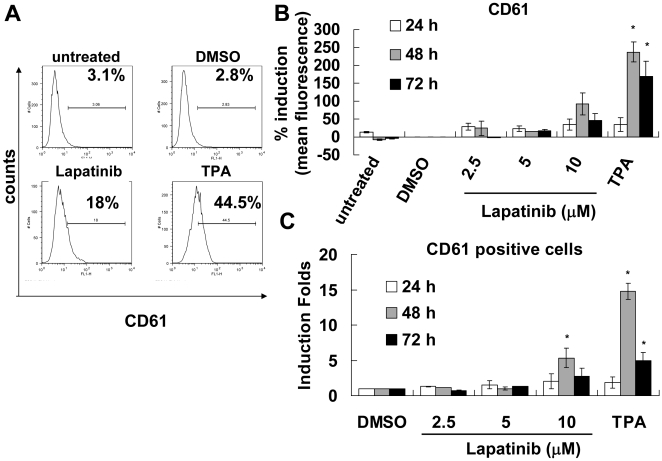
Induction of megakaryocytic differentiation by lapatinib in K562 cells. (A) After 10-µM lapatinib or 1-µM TPA treatment, K562 cells were collected, stained with anti-CD61-FITC, and the fluorescent intensity of FITC in the live cells was analyzed. The percentage of CD61 positive cells for each figure is indicated. (B) Data from separate experiments of drug exposure for 1–3 days in (A) are expressed as mean induction folds of CD61 positive cells as follows: percentage of CD61 positive cells (lapatinib or TPA)/percentage of CD61 positive cells (DMSO). (C) Data from separate experiments of lapatinib exposure for 1–3 days in (A) are expressed as percentage induction of mean fluorescence as follows: [mean fluorescence (lapatinib or TPA)/mean fluorescence (DMSO)]×100. *P<0.05 (*t*-test).

Some K562 cells were observed to exhibit giant contours, a feature typical of megakaryocytes (Fig. 3A). To validate the observed megakaryocytic differentiation, we used flow cytometry to detect surface expression of CD61, a megakaryocytic marker (Fig. 6). Lapatinib induced moderately upregulated expression of CD61 (Figs. 3A and 6). TPA was used to verify the megakaryocytic differentiation [23]. Differentiation toward the erythroid cell lineage was excluded by lack of staining with benzidine in lapatinib-treated K562 cells (data not shown).

## Discussion

In this study we demonstrated that lapatinib induced myeloid leukemia cell death in CML K562, MEG-01, AML HL-60 and NB4 cells (Figs. 1A–1E) and showed much more toxicity than their normal counterpart human CD14^+^ monocytes, or mouse bone marrow cells (Fig. 1F). The lack of cell growth in these two primary cell populations, as indicated by the decrease cell number in the untreated cells on day 2 and day 3 when compared to the robust growth kinetics of K562 cells (Fig. 1F), possibly is caused by differential sensitivity to lapatinib treatment on primary and cancer cells. Lapatinib induced both apoptosis and autophagy (Figs. 2, 3, 4, 5, and 6) in CML K562 cells, which correlated with the induction of megakaryocytic differentiation of the cells. The IC_50_ of lapatinib, as shown by MTT assay, was about 1.49 µM for K562 cells (Fig. 1). The sensitivity to lapatinib varies among different human cancer cell lines. For example, the IC_50_ ranged from 0.01 to 18.6 µM for breast, 0.057 to 11.5 µM for lung, 0.029 to 3.074 µM for head and neck, and 1.51 to 7.7 µM for colon cancer cell lines [10], [12], [24], [25]. This implies that the therapeutic window for each type of cancer needs to be determined in vivo after screening anti-cancer activity using in vitro systems.

Some recent studies have focused on autophagy and necroptosis as causes of programmed cell death [26], [27]. The differentiating features of these types of cell death in comparison with apoptosis include massive autophagic vacuolization (double-membrane vacuoles) inside the cytoplasm and the occurrence of cell death in the absence of chromosome condensation and nuclear fragmentation [16], [28], [29]. In our study, besides autophagic vacuoles, specific features of autophagic cell death also included conversion of LC-I to LC-II and involvement of autophagy-related proteins and Beclin-1 (Figs. 3 and 5). In addition, induction of autophagy by lapatinib in K562 cells included the protection of cells from lapatinib-induced cell death by an autophagy inhibitor and knockdown of autophagy-related proteins (Figs. 4 and 5). Induction of autophagy marker LC3-II in lapatinib-treated K562 cells occurred in a dose dependent manner (Fig. 3B), similar to the effect of lapatinib in HCT116 colon cancer cells [30]. Only a few articles have discussed the induction of autophagy by lapatinib, including one in which HCT116 colon cancer cells were used as the model cell system [30]. LC3-I constitutive expression is a relatively unique characteristic of K562 cells (Fig. 3) [31], which is consistent with recent studies that have noted the constitutive formation of autophagy-related precursor structures in K562 cells regardless of nutritional conditions [16], [32], [33]. Consistent with the induction of autophagy by lapatinib, we found that the pancaspase inhibitor z-VAD-fmk only weakly reduced growth inhibition by lapatinib despite an effective blockage of apoptotic cell death (Figs. 3C–3D). The autophagic marker LC3II was further increased by z-VAD-fmk when K562 cells were treated with 5 µM lapatinib (Fig. 3E), suggesting more cells underwent autophagy when the apoptotic pathway was blocked by z-VAD-fmk [34]. Unlike results reported for U937 or L929 cells [35], we did not find cytotoxicity with 20-µM z-VAD-fmk treatment alone in K562 cells (Fig. 3C). We further found that autophagy correlated with differentiation in K562 cells. This result is consistent with a similar finding in TPA-treated K562 cells [20]. Necroptosis is necrosis-like programmed cell death that does not include degradation and condensation of chromosomal DNA, similar to autophagic cell death. Recent findings suggest that receptor-interacting protein 3 (RIP3) switches TNF-induced death to receptor-interacting protein 1 (RIP1)-related necroptosis [36]. Further experiments to explore the role of RIP1 in lapatinib-induced cell death are ongoing.

We further found that lapatinib induced differentiation of K562 cells. Considerable evidence suggests that induction of cytotoxic activity and differentiation occurs with other inhibitors of EGFR, such as gefitinib and erlotinib, in both leukemia cell lines and leukemia patients [37], [38], [39], [40], [41], [42], [43], [44], [45]. Interestingly, one report discussed complete remission in a patient with acute myelogenous leukemia after treatment with erlotinib [46], which resembled the application of all trans retinoic acid (ATRA) for acute promyelocytic leukemia by changing the differentiation status.

To the best of our knowledge, the evidence that lapatinib induces three distinct cellular events is unique. The possible action scenarios might be: (1) cells progress from one cellular event to another, (2) three subpopulations of cells respond differentially to lapatinib, or (3) a switch exists between each event. Although a possible switch between apoptosis and autophgy has been proposed (Fig. 3E and discussed in [47]), this issue warrants further investigation. In addition, further experiments are required to explore whether the mechanisms of lapatinib-induced cell death in leukemia are different than the mechanisms that kill cancer cell lines that die in much lower concentrations, such as breast cancer cells with higher levels of ErbB2 expression [10], [12], [24], [25].

The potential targets of lapatinib or off-target effects of lapatinib in leukemia need to be further elucidated, since there was no evidence of expression of EGFR or ErbB2 in K562 cells [48], [49]. A few studies have reported the expression of ErbB receptor members in a subset of lymphoid cells from patients with CML or acute lymphoblastic leukemia [48], [50], [51]. Our preliminary results and other studies have shown that ErbB2 transcripts, but not EGFR transcripts, were detectable in leukemia cell lines other than K562 (data not shown) [43], [49]. Interestingly, in HL-60, ErbB2 transcript was detectable, but ErbB2 protein was undetectable [43]. The expression profiles of ErbB family members and their correlations with lapatinib sensitivity remain to be investigated. Along the same lines, ErbB2 and IGF-1R heterodimers contributed to trastuzumab resistance in breast cancer cells [5], [6]. Additionally, the IGF-1R autocrine loop is an important survival signal in leukemia [52], and IGF-1R signaling synergistically amplifies Abl receptor tyrosine kinase aberrant forms, one of the most common mutations in leukemia [53]. In an effort to further dissect the mechanisms of the cytotoxic effects of lapatinib in K562 cells, we attempted to investigate the kinetics of BCR-Abl expression by Western blot; however, we found the results to be complicated: both BCR-Abl expression and phosphotyrosine of BCR-Abl were upregulated on day 1 but downregulated on day 2 in lapatinib-treated K562 cells (data not shown). Therefore, we will continue to study potential targets of lapatinib in CML K562 cells.

In conclusion, we demonstrated induction of autophagy, apoptosis, and differentiation of K562 cells upon lapatinib treatment. Apoptosis was likely induced by a caspase-dependent pathway and autophagic cell death was likely induced via an ATG6-dependent pathway. These findings suggest that lapatinib may have potential for the treatment of leukemia.
